# A prognostic nomogram for neuroblastoma in children

**DOI:** 10.7717/peerj.7316

**Published:** 2019-07-11

**Authors:** Xiaozhi Li, Yutong Meng

**Affiliations:** 1 Department of Neurosurgery, Shengjing Hospital of China Medical University, Shenyang, China; 2 Department of Stomatology, Shengjing Hospital of China Medical University, Shenyang, China

**Keywords:** Neuroblastoma, TARGET, Nomogram, Prognosis

## Abstract

**Introduction:**

Neuroblastoma is one of the most common extracranial solid tumors in children, which accounts for about 7–10% in children’s tumors. The prognosis group of patients with neuroblastoma could not only improve the efficacy of high-risk patients, but also reduce the effects of drug complications for surviving patients.

**Material and Methods:**

Patients diagnosed with neuroblastoma between 1986 and 2012 were selected form the TARGET database. The nomogram was built with potential risk factors based on COX regression analysis. The precision of the 3-year and 5-year survival of the nomograms was evaluated by the area under receiver operating characteristic (ROC) curve (AUC).

**Results:**

A total of 757 child neuroblastoma patients were selected from the TARGET database. Univariate analysis showed that age of diagnosis (>520 day), race of American Indian or Alaska Native, stage 4 in International Neuroblastoma Staging System (INSS), *MYCN* status, DNA ploidy, and high mitosis-karyorrhexis index were associated with overall survival (OS). Multivariate analysis showed age of diagnosis (>520 day), stage 4 in INSS and DNA ploidy were independent risk factors of OS. The concordance index (C-index) of the nomogram was 0.704 (95% CI [0.686–0.722]) in the training cohort while the C-index in the validation cohort was 0.672 (95% CI [0.644–0.700]). AUC values of ROC curves for 3-year OS and 5-year OS in the training cohort were 0.732 and 0.772, respectively. The nomogram performed better compared with INSS staging system, tumor histology and children’s oncology group (COG) risk group with C-indexes of 0.662 (95% CI [0.648–0.676]), 0.637 (95% CI [0.622–0.652]) and 0.651 (95% CI [0.637–0.665]), respectively.

**Conclusions:**

The nomogram showed stronger predictive power than the INSS staging system, tumor histology and COG risk group. Precise estimates of the prognosis of childhood neuroblastoma might help doctors make better treatment decisions.

## Introduction

Neuroblastoma is one of the most common extracranial solid tumor in children, which accounts for about 7–10% in children’s tumors (Ahmed et al., 2017; Kholodenko & Kalinovsky, 2018; Nakagawara et al., 2018). Neuroblastoma originates from nerve sympathetic cells and can occur in sympathetic neuronal cells of embryonic neural crest cells. It can arise anywhere along the sympathetic nervous system chain, including the abdomen, adrenal gland, chest, neck, and pelvis, and is often transferred to the parts such as focal lymph node, marrow, skeleton, and liver (Mlakar et al., 2017). Early diagnosis of neuroblastoma is difficult, with a high degree of malignancy and easy to metastasis. The cancer mortality rate of neuroblastoma in children is just fall behind leukemia and brain tumors, which accounts for about 11% (Becker & Wilting, 2018). The prognosis of patients with neuroblastoma is quite variant. The prognosis group of patients with neuroblastoma was classify in to high-risk patients and low-risk patients, which could not only improve the efficacy of high-risk patients, but also reduce the effects of drug complications for surviving patients. Therefore, it is necessary to analyze the prognosis of patients with neuroblastoma.

Clinically, prognostic factors of neuroblastoma include factors such as age of diagnose, International Neuroblastoma Staging System (INSS) stage, *MYCN* status, and tumor histology (Whittle et al., 2017). *MYCN* is a prognostic related gene of neuroblastoma. Approximately 25% of patients presented with *MYCN* gene amplification which was regarded as a biomarker for poor prognosis (Zhou et al., 2018). Levels of DNA ploidy have been found to be one of significant prognostic factors. Patients with DNA aneuploid neuroblastoma died less frequently than those with DNA diploid tumors (Eckschlager et al., 1996). The INSS staging system is widely used in the clinical staging of primary organs and metastases in neuroblastoma. In addition, the children’s oncology group (COG) risk group (London et al., 2017) is a commonly used system for therapeutic decision-making reference, which dividing patients with neuroblastoma into low-risk, intermediate-risk, and high-risk groups. The COG risk group imports factors such as INSS stage, age of diagnose, *MYCN*, tumor histology, and DNA ploidy status.

The therapeutically applicable research to generate effective treatment (TARGET) database is a project containing experimental and clinical materials of a number of caners of children. TARGET project is affiliated to the Center for Cancer Genomics of the National Cancer Institute and is aimed to use data to guide the development of effective and less toxic therapies. In this study, we collected and analyzed the information of child neuroblastoma patients from the TARGET database and explored possible prognostic nomogram for more accurate assessment of the prognosis of patients of children with neuroblastoma.

## Materials and Methods

### Data source and eligibility criteria

We downloaded clinical data of patients with neuroblastoma from the TARGET project database (https://ocg.cancer.gov/) (Pugh et al., 2013; Zhou et al., 2019). The inclusion criterion was that the patients were diagnosed with neuroblastoma from 1986 to 2012. Exclusion criteria were as follows: unknown age of diagnosis, uncertain gender, undetermined race, uncertain INSS stage, uncertain *MYCN* amplification status, unknown DNA ploidy status, undetermined tumor histology, unclear pathological histology results, uncertain mitosis-karyorrhexis index (MKI), unknown COG risk group, and the age is over 18 years old.

The categorical measurements were described as counts and percentages, and the continuous measurements were presented as mean and range. The *t-*test was used to continuous measurements, while the Chi-square for compare ones. *P* < 0.05 was considered statistically significant. Overall survival (OS) were used as primary end points. OS was defined as the interval from the time of diagnosis to death or last follow-up regardless of death cause. Optimal cutoff value of age was determined by “survminer” package of R software (Kassambara et al., 2019). The nomogram was built with potential risk factors based on COX regression analysis in the training cohort. concordance index (C-index) was used to estimate predictive performance of the nomogram. The larger the C-index, the more accurate the model prediction. The C-index of the nomogram was compared with the C-index of INSS staging system, tumor histology and COG risk group. Calibration plots were used to compare the observed and predicted probabilities for the nomogram. The precision of the 3-year and 5-year survival of the nomograms was evaluated by the area under ROC (Receiver operating characteristic) curve (AUC). The flow diagram is shown in Fig. S1.

## Results

### Patient characteristics

The original cohort with a total of 1,119 patients was involved. A total of 757 patients complied with inclusion criteria. The median OS was 1,668 days (range 10–5,216 days). The 3-year and 5-year OS rates were 75.54% and 44.39%, respectively. We used R software (3.5.2) as our statistical analysis tool. Optimal cutoff value of age of diagnosis was 520 days. 70% of all patients were randomly selected to form the training cohort for the construction of nomogram while the rest 30% patients served as the validation cohort. The demographics and clinicopathologic characteristics of training cohort (*n* = 532), validation cohort (*n* = 225) and all patients (*n* = 757) are shown in Table 1.

**Table 1:** Demographics and clinicopathologic characteristics of patients with neuroblastoma of children.

Demographics or characteristic	Training cohort *n* = 532 (%)	Validation cohort *n* = 225 (%)	*P-*value
**Sex**			0.976
Female	224 (42.11)	95 (42.22)	
Male	308 (57.89)	130 (57.78)	
**Age, days**			0.719
≤520	175 (32.89)	71 (31.56)	
>520	357 (67.11)	154 (68.44)	
**Race**			0.960
White	443 (83.27)	183 (81.33)	
Native Hawaiian or other Pacific Islander	6 (1.13)	2 (0.89)	
Black or African American	66 (12.41)	32 (14.22)	
Asian	15 (2.82)	7 (3.11)	
American Indian or Alaska Native	2 (0.28)	1 (0.44)	
**INSS stage**			0.911
Stage 1	49 (9.21)	23 (10.22)	
Stage 2	37 (6.95)	18 (8.00)	
Stage 3	53 (9.96)	23 (10.22)	
Stage 4	393 (73.87)	161 (71.56)	
***MYCN* status**			0.551
** **Not amplified	378 (71.05)	155 (68.89)	
Amplified	154 (28.95)	70 (31.11)	
**Ploidy**			0.864
Diploid (DI=1)	195 (36.65)	81 (36.00)	
Hyperdiploid (DI>1)	337 (63.35)	144 (64.00)	
**MKI**			0.819
Low	214 (40.23)	85 (37.78)	
Intermediate	158 (29.7)	70 (31.11)	
High	160 (30.08)	70 (31.11)	
**Histology**			0.514
Unfavorable	368 (69.17)	161 (71.56)	
Favorable	164 (30.83)	64 (28.44)	
**COG risk group**			0.155
Low risk	105 (19.74)	34 (15.11)	
Intermediate risk	58 (10.90)	33 (14.67)	
High risk	369 (69.36)	158 (70.22)	

### Prognostic factors of OS in the training cohort

To analyze prognostic factors of OS, we used the univariate analysis and multivariate analysis. As shown in Table 2, univariate analysis showed that age of diagnosis (>520 days), race of American Indian or Alaska Native, stage 4 in INSS, *MYCN* status, DNA ploidy, high MKI were associated with OS. Meanwhile, multivariate analysis showed age of diagnosis (>520 days), stage 4 in INSS and DNA ploidy were independent risk factors of OS.

**Table 2:** Univariate analysis and multivariate analysis in the training cohort.

Demographics or characteristic	No.	Univariate analysis		Multivariate analysis	
		HR (95%CI)	*P*	HR (95%CI)	*P*
**Sex**					
Female	224				
Male	308	0.916 [0.682–1.229]	0.558	0.798 [0.588–1.084]	0.148
**Age, days**					
≤520	175				
>520	357	4.720 [2.963–7.519]	0.000	1.750 [1.041–2.944]	0.035
**Race**					
White	443				
Native Hawaiian or other Pacific Islander	6	1.126 [0.279–4.548]	0.868	1.122 [0.273–4.609]	0.873
Black or African American	66	0.912 [0.578–1.439]	0.691	0.787 [0.495–1.251]	0.311
Asian	15	0.801 [0.297–2.162]	0.662	0.711 [0.258–1.958]	0.509
American Indian or Alaska Native	2	5.639 [1.393–22.829]	0.015	3.486 [0.829–14.666]	0.088
**INSS Stage**					
Stage 1	49				
Stage 2	37	3.061 [0.277–33.779]	0.361	2.148 [0.191–24.170]	0.536
Stage 3	53	4.679 [0.523–41.901]	0.168	2.926 [0.317–26.993]	0.344
Stage 4	354	34.724 [4.860–248.092]	0.000	17.073 [2.257–129.178]	0.006
Stage 4S	39	4.751 [0.494–45.708]	0.177	4.633 [0.481–44.638]	0.185
***MYCN* status**					
Not Amplified	378				
Amplified	154	1.935 [1.434–2.612]	0.000	1.226 [0.843–1.782]	0.286
**Ploidy**					
Diploid (DI = 1)	195				
Hyperdiploid (DI > 1)	337	0.410 [0.306–0.550]	0.000	0.641 [0.470–0.876]	0.005
**MKI**					
Low	214				
Intermediate	158	1.385 [0.950–2.021]	0.090	0.884 [0.595–1.314]	0.543
High	160	2.176 [1.532–3.090]	0.000	1.094 [0.716–1.673]	0.677

### Prognostic nomogram for OS

**Figure 1:**
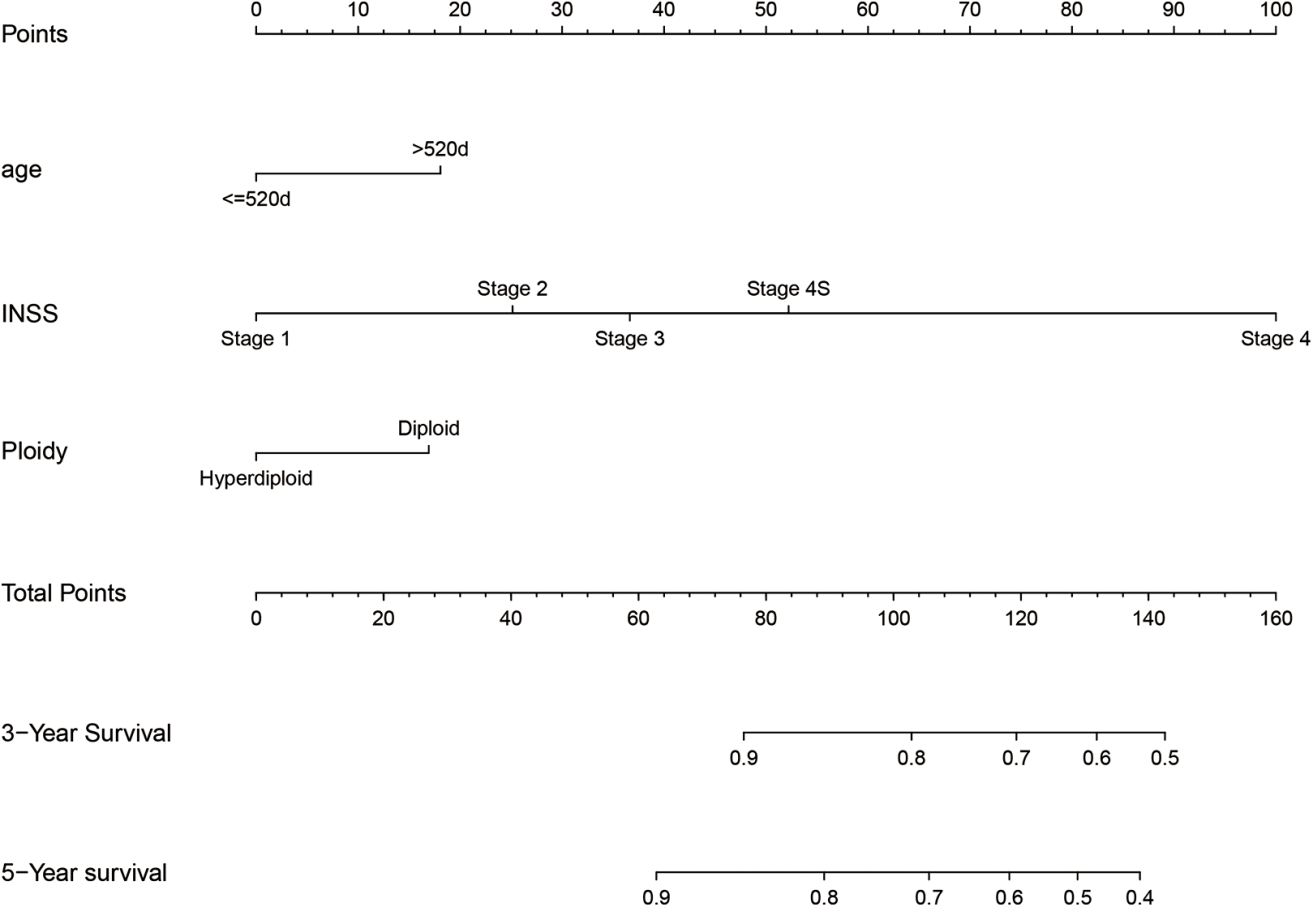
Nomogram to predict the probability of 3-year OS and 5-year OS.

**Figure 2:**
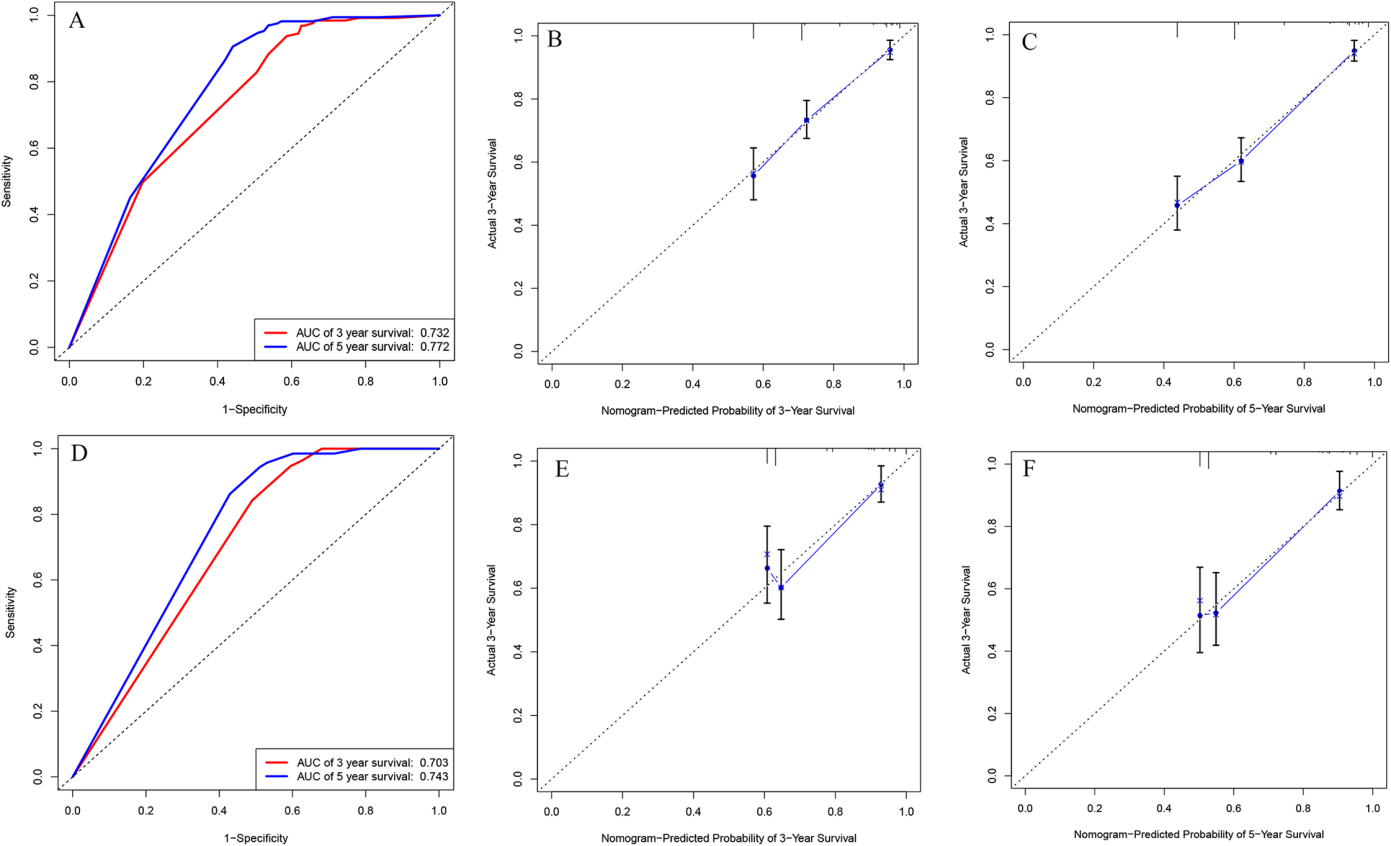
ROC curves and calibration plots of the nomogram in training and validation cohorts. (A) ROC curves for discrimination in the training set. (B) Calibration plot of observed and predicted probabilities for the nomogram in the training set at 3 years. (C) Calibration plot of observed and predicted probabilities for the nomogram in the training set at 5 years. (D) ROC curves for discrimination in the validation set. (E) Calibration plot of observed and predicted probabilities for the nomogram in the validation set at 3 years. (F) Calibration plot of observed and predicted probabilities for the nomogram in the validation set at 5 years.

We integrated the factors including age of diagnose, INSS stage and DNA ploidy to form the prognostic nomogram (Fig. 1). The C-index of the nomogram was 0.704 (95% CI [0.686–0.722]) in the training cohort while the C-index in the validation cohort was 0.672 (95% CI [0.644–0.700]). As shown in Fig. 2, the AUC values of ROC curves for 3-year OS and 5-year OS in the training cohort were 0.732 and 0.772, respectively. Besides, the AUC values of ROC curve for 3-year OS and 5-year OS in the validation cohort were 0.703 and 0.743, respectively. The calibration plots for the probability of 3-year OS and 5-year OS indicated no apparent departure form ideal line with optimal agreement between prediction by nomogram and observation in both training cohort and validation cohort.

**Figure 3:**
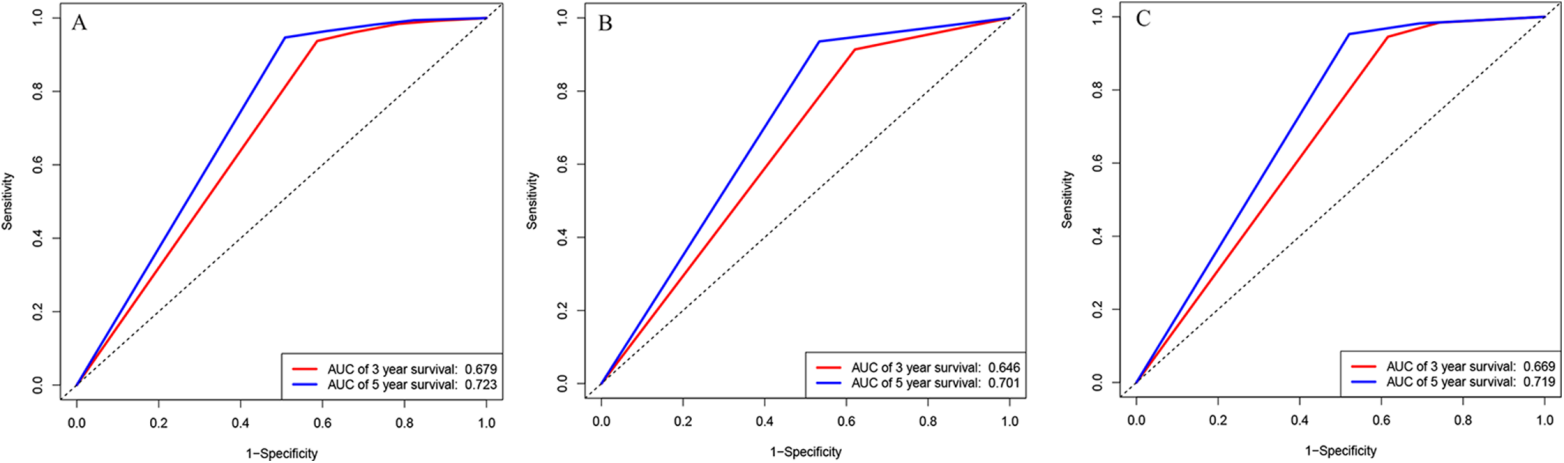
ROC curves. ROC curves of (A) INSS staging system, (B) histology groups of Shimada system, and (C) COG staging system in training cohort.

We also analyzed the ROC curves and calculated C-index for the prognostic ability of INSS staging system, tumor histology, and COG risk group. As shown in Fig. 3, the AUC values of ROC curves for 3-year OS and 5-year OS in the training cohort were 0.679 and 0.732 in INSS staging system, respectively. The AUC values of ROC curves for 3-year OS and 5-year OS in the training cohort were 0.646 and 0.701 in tumor histology system, respectively. The AUC values of ROC curves for 3-year OS and 5-year OS in the training cohort were 0.669 and 0.719 in COG risk group. The C-indexes for INSS staging system, tumor histology and COG risk group were 0.662 (95% CI [0.648–0.676]), 0.637 (95% CI [0.622–0.652]) and 0.651 (95% CI [0.637–0.665]) in the training cohort, respectively. While C-indexes for INSS staging system, tumor histology and COG risk group were 0.649 (95% CI [0.630–0.668]), 0.641 (95% CI [0.621–0.661) and 0.663 (95% CI [0.645–0.681]) in the validation cohort, respectively.

### Survival curves for prognostic factors

**Figure 4:**
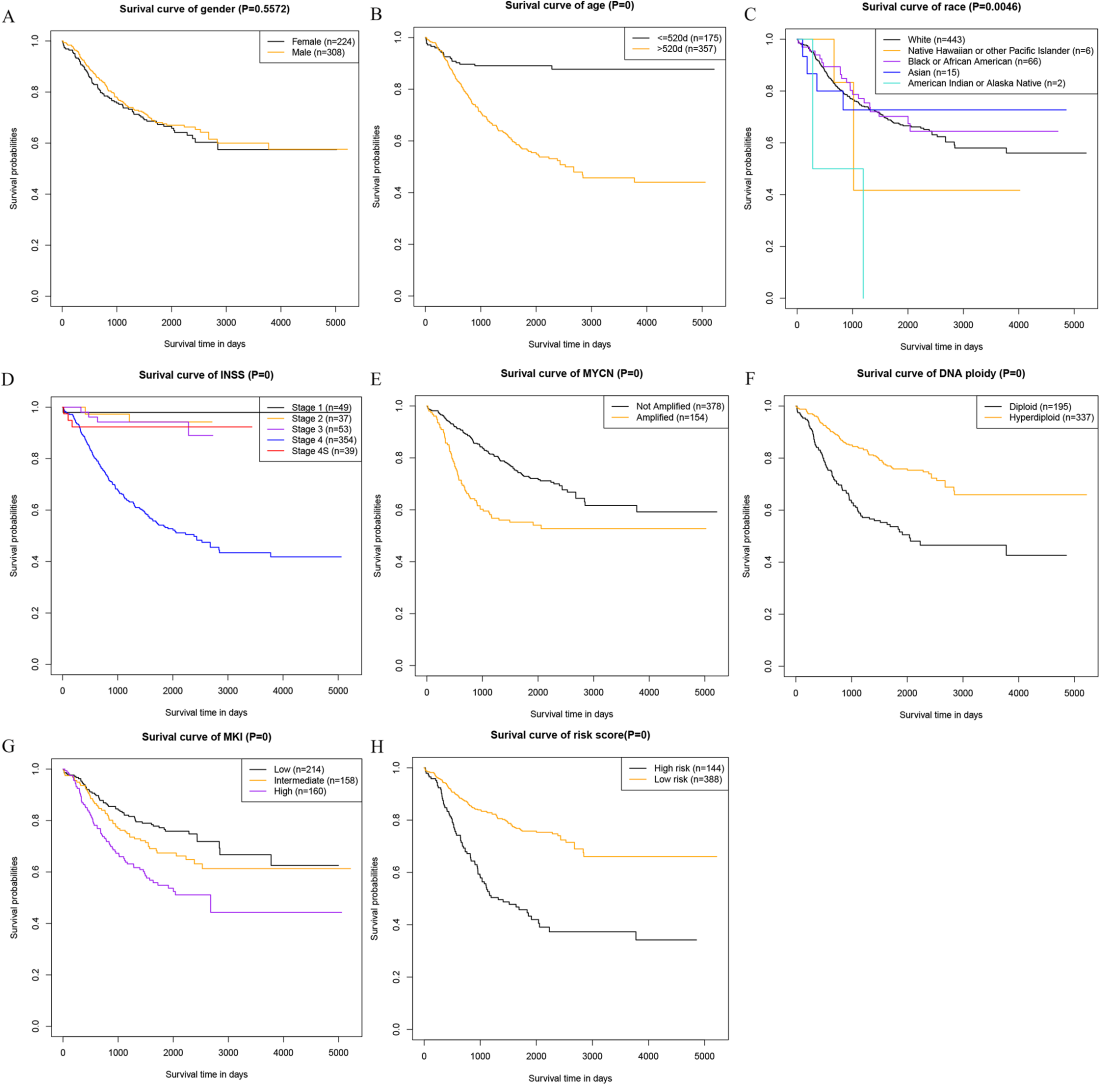
Kaplan–Meier survival curves of the training cohort. (A) Kaplan–Meier survival curves of gender. (B) Kaplan–Meier survival curves of age. (C) Kaplan–Meier survival curves of race. (D) Kaplan–Meier survival curves INSS. (E) Kaplan–Meier survival curves of *MYCN*. (F) Kaplan–Meier survival curves Ploidy. (G) Kaplan–Meier survival curves MKI. (H) Kaplan–Meier survival curves risk score.

At last, we analyzed the correlation between the prognostic factors in the nomogram and the OS, and drew the survival curves (Fig. 4). The description of risk levels in the training cohort is shown in Table S3. We found that age, race, INSS staging system, *MYCN*, DNA ploidy status, MKI, and calculated risk scores were associated with overall survival.

### Clinical use of the nomogram

For example, one case of the cohort whose age was 1,758 days (>520 days) was identified as stage 4 in INSS and hyperdiploid status. The estimated point was about 18 + 100 + 0 =118. The estimated 3-year survival rate and 5-year survival rate was about 80% and 72%, respectively. Actually, this patient died at the age of 2,846 days (7.80 years).

## Discussion

Our study found that age of diagnosis (>520 days), stage 4 in INSS and DNA ploidy were independent prognostic risk factors for neuroblastoma in children. Then the prognostic nomogram was constructed based on age of diagnose, INSS stage, and DNA ploidy status to predict 3-year OS and 5-year OS. The nomogram can provide a relatively accurate estimate of the prognosis of patients. There are many reports that the nomogram has higher accuracy than the conventional tumor staging system (He et al., 2018; Wang et al., 2018; Wang et al., 2013). The nomogram showed stronger predictive power than the INSS staging system, tumor histology, and COG risk group.

The nomogram is mainly used to predict the relevant conditions of the disease. In terms of tumors, the nomogram is mainly used for prognostic models such as tumor patient mortality, lymph node metastasis, and complications (Jiang et al., 2019; Wei et al., 2019). The nomogram is a new form of disease risk estimation that can aid clinical decision making (Botticelli et al., 2019). Studies based on the Surveillance, Epidemiology, and End Results Program (SEER) database showed that gender had no significant difference in the OS of neuroblastoma in children (Stokes et al., 2015). These results were consistent with our findings. Zhao reported clinical data of 155 children with neuroblastoma under the age of 18. It was found that the patients had 3-year and 5-year overall survival rate of 96.2% and 94.1%, and compared with 12–18 months, 12 month-old-children had a better prognosis. *MYCN* amplification, elevated LDH was associated with poor prognosis (Zhao et al., 2017). Campbell reviewed 4,672 patients with neuroblastoma and found that patients with *MYCN* amplification had lower event-free survival and lower overall survival (Campbell et al., 2017). Moreover, studies had shown that *MYCN* amplification in serum of patients with neuroblastoma was also associated with overall patient survival (Yagyu et al., 2016). Neuroblastoma patients with overexpressing *MYCN* and *MYCN* protein had lower survival rates (Bansal et al., 2017; Wang et al., 2015). In addition to *MYCN* gene abnormalities, deletion of 11q was also associated with prognosis in patients with neuroblastoma (Caren et al., 2010). Based on the SEER database, it was found that stage 4 disease, unfavorable DNA ploidy, *MYCN* gene amplification decreased the 5-year survival rates (Coughlan et al., 2017), while age, tumor location, stage are independent prognostic factors for neuroblastoma (Gutierrez et al., 2007).

The International Neuroblastoma Staging System staging system is widely used in patients with neuroblastoma, which is also a common prognostic indicator (Bansal et al., 2017; Tan et al., 2012). The International Neuroblastoma Pathology Classification (INPC) classifies neuroblastoma into the favorable histology (FH) group and the unfavorable histology (UFH) group. The FH group had an earlier onset and a good prognosis, while the UFH group had a poor prognosis (Nakazawa et al., 2015). The COG risk group was established in 1998 for prognosis of neuroblastoma. Age of diagnose, INSS stage, INPC, *MYCN* status, and DNA ploidy status are included in the COG risk group. After the application of the COG risk group, the survival of patients with neuroblastoma is greatly improved (Elzomor et al., 2018).

The optimal cutoff of age in this study was determined by the “survminer” package of R software using maximally selected rank statistics (the P-age curve is shown in Fig. S2). The cutoff was younger than that suggested by London et al. (2005) probably because of selection of cases or inclusion criteria.

However, there are also many limitations in this study. For example, the different treatment options of individuals and secondary cancer may influence the effectiveness of model. There are many breakthroughs in the treatment of neuroblastoma in recent years (Tolbert & Matthay, 2018), which may also have survival impact affecting the study results. Moreover, adding more updated data to the study can make the results more accurate. Other potential factors such as TERT rearrangements, ATRX mutations, CCND1 amplification may also perform well in prognosis of neuroblastoma. In addition, due to the limitations of the collected case data, the study case cannot include population characteristics in all regions, which may affect the nomogram to reflect all population characteristics.

## Conclusion

Analysis of clinical data based on big data is an important source of clinical prognostic indicators. The vast majority of current clinical data analysis is based on the SEER database. The TARGET database is part of the National Cancer Institute project and its clinical data is rarely reported. As we known, we analyzed the primary data of childhood neuroblastoma for the first time. Based on the patients’ primary clinical data, the prognostic nomogram of the prognosis of childhood neuroblastoma was established, whose accuracy was higher than the INSS staging system, the INPC, and the COG risk group. Precise estimates of the prognosis of childhood neuroblastoma might help doctors assess the patients’ actual condition, select appropriate treatment options, and develop better follow-up plans.

## Supplemental Information

10.7717/peerj.7316/supp-1 Supplemental Information 1 Dataset S1. Raw data exported from the TARGET database: gender, race, age at diagnosis, INSS stage, MYCN status, ploidy status, MKI status, histology status, and COG risk group.

10.7717/peerj.7316/supp-2 Supplemental Information 2 Dataset S2. Raw data exported from the TARGET database: gender, race, age at diagnosis, INSS stage, MYCN status, ploidy status, MKI status, histology status, and COG risk group.

10.7717/peerj.7316/supp-3 Supplemental Information 3 Coefficients for calculating risk score.

10.7717/peerj.7316/supp-4 Supplemental Information 4 Flow diagram.

10.7717/peerj.7316/supp-5 Supplemental Information 5 Log_10_(*P*) of different cutoff of age. (A) Log_10_(*P*)-age plot. (B) Close-up view of Fig. S2A.

## Additional Information and Declarations

### Data Availability

The following information was supplied regarding data availability: The raw measurements are available in Tables S1 and S2.

## References

[ref-1] Ahmed AA, Zhang L, Reddivalla N, Hetherington M (2017). Neuroblastoma in children: update on clinicopathologic and genetic prognostic factors. Pediatric Hematology and Oncology.

[ref-2] Bansal D, Totadri S, Chinnaswamy G, Agarwala S, Vora T, Arora B, Prasad M, Kapoor G, Radhakrishnan V, Laskar S, Kaur T, Rath GK, Bakhshi S (2017). Management of neuroblastoma: ICMR consensus document. Indian Journal of Pediatrics.

[ref-3] Becker J, Wilting J (2018). WNT signaling, the development of the sympathoadrenal-paraganglionic system and neuroblastoma. Cellular and Molecular Life Sciences.

[ref-4] Botticelli A, Salati M, Di Pietro FR, Strigari L, Cerbelli B, Zizzari IG, Giusti R, Mazzotta M, Mazzuca F, Roberto M, Vici P, Pizzuti L, Nuti M, Marchetti P (2019). A nomogram to predict survival in non-small cell lung cancer patients treated with nivolumab. Journal of Translational Medicine.

[ref-5] Campbell K, Gastier-Foster JM, Mann M, Naranjo AH, Van Ryn C, Bagatell R, Matthay KK, London WB, Irwin MS, Shimada H, Granger MM, Hogarty MD, Park JR, DuBois SG (2017). Association of *MYCN* copy number with clinical features, tumor biology, and outcomes in neuroblastoma: a report from the children’s oncology group. Cancer.

[ref-6] Caren H, Kryh H, Nethander M, Sjoberg R-M, Trager C, Nilsson S, Abrahamsson J, Kogner P, Martinsson T (2010). High-risk neuroblastoma tumors with 11q-deletion display a poor prognostic, chromosome instability phenotype with later onset. Proceedings of the National Academy of Sciences of the United States of America.

[ref-7] Coughlan D, Gianferante M, Lynch CF, Stevens JL, Harlan LC (2017). Treatment and survival of childhood neuroblastoma: evidence from a population-based study in the United States. Pediatric Hematology and Oncology.

[ref-8] Eckschlager T, Pilat D, Kodet R, Dahbiova R, Stankova J, Jasinska J, Hrusak O (1996). DNA ploidy in neuroblastoma. Neoplasma.

[ref-9] Elzomor H, Ahmed G, Elmenawi S, Elkinaai N, Refaat A, Soliman S, Abdelwahab MA, Zaghloul MS, Fawzy M (2018). Survival outcome of intermediate risk neuroblastoma at Children Cancer Hospital Egypt. Journal of the Egyptian National Cancer Institute.

[ref-10] Gutierrez JC, Fischer AC, Sola JE, Perez EA, Koniaris LG (2007). Markedly improving survival of neuroblastoma: a 30-year analysis of 1,646 patients. Pediatric Surgery International.

[ref-11] He C, Zhang Y, Cai Z, Lin X, Li S (2018). Overall survival and cancer-specific survival in patients with surgically resected pancreatic head adenocarcinoma: a competing risk nomogram analysis. Journal of Cancer.

[ref-12] Jiang H-H, Dong X-L, Tang X, Li A-J, Chang Y, Li H-G, Chen Y, Zhang Z-Y, Tang E-J, Lin M-B (2019). Nomogram for predicting risk of intestinal complications after colorectal cancer surgery. Medical Science Monitor.

[ref-32] Kassambara A, Kosinski M, Biecek P, Fabian S (2019). Survminer: drawing survival curves using ‘ggplot2’. https://cran.r-project.org/web/packages/survminer/index.html.

[ref-13] Kholodenko IV, Kalinovsky DV (2018). Neuroblastoma origin and therapeutic targets for immunotherapy. Journal of Immunology Research.

[ref-14] London WB, Bagatell R, Weigel BJ, Fox E, Guo D, Van Ryn C, Naranjo A, Park JR (2017). Historical time to disease progression and progression-free survival in patients with recurrent/refractory neuroblastoma treated in the modern era on children’s oncology group early-phase trials. Cancer.

[ref-15] London WB, Castleberry RP, Matthay KK, Look AT, Seeger RC, Shimada H, Thorner P, Brodeur G, Maris JM, Reynolds CP, Cohn SL (2005). Evidence for an age cutoff greater than 365 days for neuroblastoma risk group stratification in the children’s oncology group. Journal of Clinical Oncology.

[ref-16] Mlakar V, Jurkovic Mlakar S, Lopez G, Maris JM, Ansari M, Gumy-Pause F (2017). 11q deletion in neuroblastoma: a review of biological and clinical implications. Molecular Cancer.

[ref-17] Nakagawara A, Li Y, Izumi H, Muramori K, Inada H, Nishi M (2018). Neuroblastoma. Japanese Journal of Clinical Oncology.

[ref-18] Nakazawa A, Haga C, Ohira M, Okita H, Kamijo T, Nakagawara A (2015). Correlation between the international neuroblastoma pathology classification and genomic signature in neuroblastoma. Cancer Science.

[ref-19] Pugh TJ, Morozova O, Attiyeh EF, Asgharzadeh S, Wei JS, Auclair D, Carter SL, Cibulskis K, Hanna M, Kiezun A, Kim J, Lawrence MS, Lichenstein L, McKenna A, Pedamallu CS, Ramos AH, Shefler E, Sivachenko A, Sougnez C, Stewart C, Ally A, Birol I, Chiu R, Corbett RD, Hirst M, Jackman SD, Kamoh B, Khodabakshi AH, Krzywinski M, Lo A, Moore RA, Mungall KL, Qian J, Tam A, Thiessen N, Zhao Y, Cole KA, Diamond M, Diskin SJ, Mosse YP, Wood AC, Ji L, Sposto R, Badgett T, London WB, Moyer Y, Gastier-Foster JM, Smith MA, Guidry Auvil JM, Gerhard DS, Hogarty MD, Jones SJM, Lander ES, Gabriel SB, Getz G, Seeger RC, Khan J, Marra MA, Meyerson M, Maris JM (2013). The genetic landscape of high-risk neuroblastoma. Nature Genetics.

[ref-20] Stokes WA, Camilon PR, Banglawala SM, Nguyen SA, Harvey R, Vandergrift WA, Schlosser RJ (2015). Is sex an independent prognostic factor in esthesioneuroblastoma?. American Journal of Rhinology & Allergy.

[ref-21] Tan C, Sabai SM, Tin AS, Quah TC, Aung L (2012). Neuroblastoma: experience from National University Health System, Singapore (1987–2008). Singapore Medical Journal.

[ref-22] Tolbert VP, Matthay KK (2018). Neuroblastoma: clinical and biological approach to risk stratification and treatment. Cell and Tissue Research.

[ref-23] Wang Y, Li J, Xia Y, Gong R, Wang K, Yan Z, Wan X, Liu G, Wu D, Shi L, Lau W, Wu M, Shen F (2013). Prognostic nomogram for intrahepatic cholangiocarcinoma after partial hepatectomy. Journal of Clinical Oncology.

[ref-24] Wang LL, Teshiba R, Ikegaki N, Tang XX, Naranjo A, London WB, Hogarty MD, Gastier-Foster JM, Look AT, Park JR, Maris JM, Cohn SL, Seeger RC, Asgharzadeh S, Shimada H (2015). Augmented expression of MYC and/or *MYCN* protein defines highly aggressive MYC-driven neuroblastoma: a children’s oncology group study. British Journal of Cancer.

[ref-25] Wang C, Yang C, Wang W, Xia B, Li K, Sun F, Hou Y (2018). A prognostic nomogram for cervical cancer after surgery from SEER database. Journal of Cancer.

[ref-26] Wei S, Zang J, Jia Y, Chen A, Xie Y, Huang J, Li Z, Nie G, Liu H, Liu F, Gao W (2019). A gene-related nomogram for preoperative prediction of lymph node metastasis in colorectal cancer. Journal of Investigative Surgery.

[ref-27] Whittle SB, Smith V, Doherty E, Zhao S, McCarty S, Zage PE (2017). Overview and recent advances in the treatment of neuroblastoma. Expert Review of Anticancer Therapy.

[ref-28] Yagyu S, Iehara T, Tanaka S, Gotoh T, Misawa-Furihata A, Sugimoto T, London WB, Hogarty MD, Teramukai S, Nakagawara A, Hiyama E, Maris JM, Hosoi H (2016). Serum-based quantification of *MYCN* gene amplification in young patients with neuroblastoma: potential utility as a surrogate biomarker for neuroblastoma. PLOS ONE.

[ref-29] Zhao J, Pan C, Xu M, Zhou M, Gao YJ, Hu WT, Tang JY (2017). Long-term follow-up of neuroblastoma in children less than 18 months of age. Zhonghua Er Ke Za Zhi.

[ref-30] Zhou K, Li X-L, Pan J, Xu J-Z, Wang J (2018). Analysis of the risk factor for the poor prognosis of localized neuroblastoma after the surgical. Medicine.

[ref-31] Zhou J, Wang J, Hong B, Ma K, Xie H, Li L, Zhang K, Zhou B, Cai L, Gong K (2019). Gene signatures and prognostic values of m6A regulators in clear cell renal cell carcinoma—a retrospective study using TCGA database. Aging.

