# Supplementary material for: A prognostic nomogram for neuroblastoma in children
Source: PeerJ. 2019 Jul 11;7:e7316. doi: 10.7717/peerj.7316 (PMC6626656; doi:10.7717/peerj.7316)
Supplement: Supplemental Information 3 [file peerj-07-7316-s003.doc]

| **Table S3. Coefficients for calculating risk score** | |
| --- | --- |
| **Item** | **Coefficient** |
| Age |  |
| age<=520d | 0 |
| age>520d | 0.5150 |
| INSS |  |
| Stage 1 | 0 |
| Stage 2 | 0.7812 |
| Stage 3 | 1.1098 |
| Stage 4 | 2.9159 |
| Stage 4S | 1.5537 |
| Ploidy |  |
| Diploid (DI=1) | 0 |
| Hyperdiploid (DI>1) | -0.4826 |
