# Supplementary figures and images for: A prognostic nomogram for neuroblastoma in children
Source: PeerJ. 2019 Jul 11;7:e7316. doi: 10.7717/peerj.7316 (PMC6626656; doi:10.7717/peerj.7316)

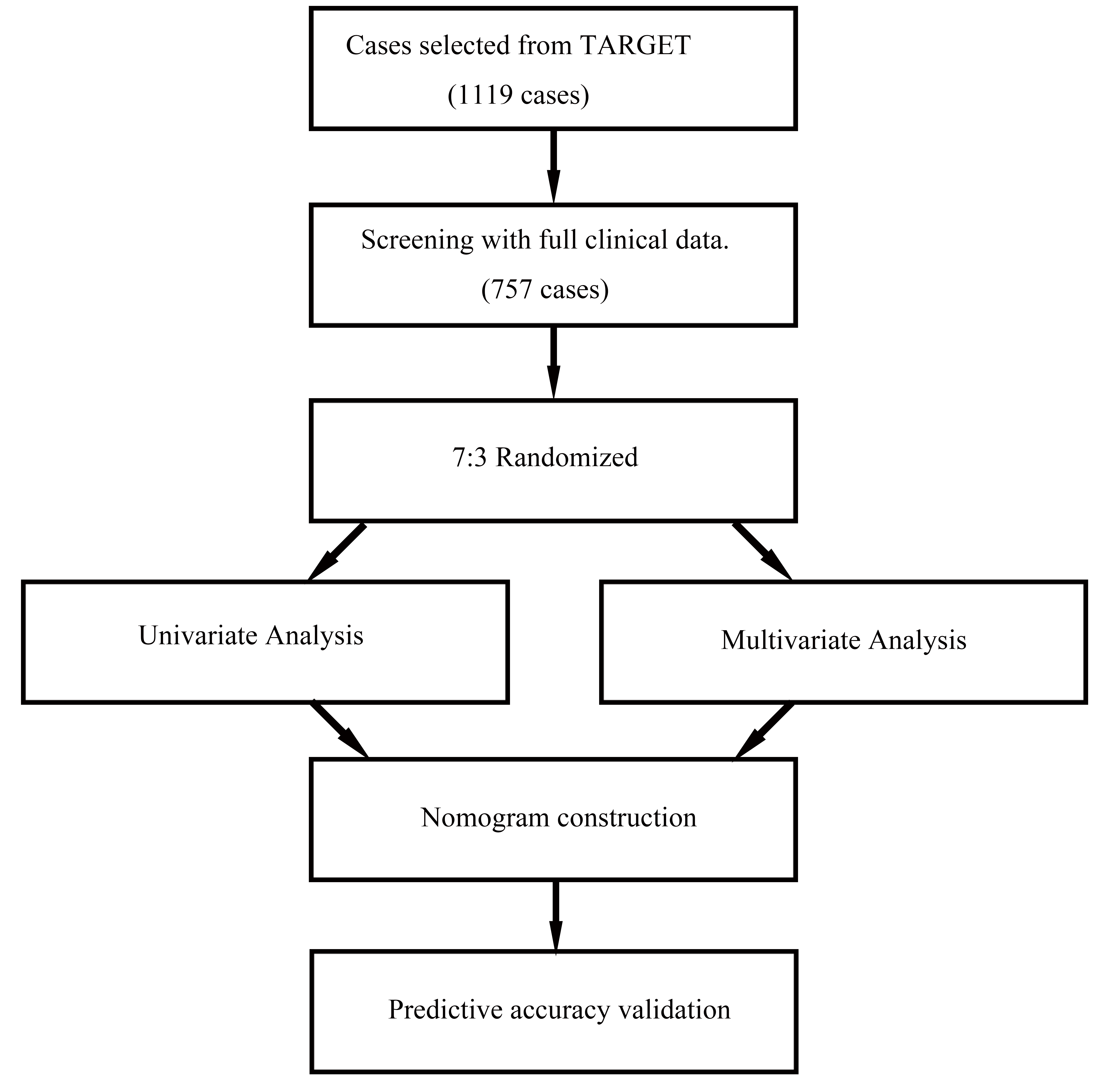

Supplement: Supplemental Information 4 [file peerj-07-7316-s004.png]

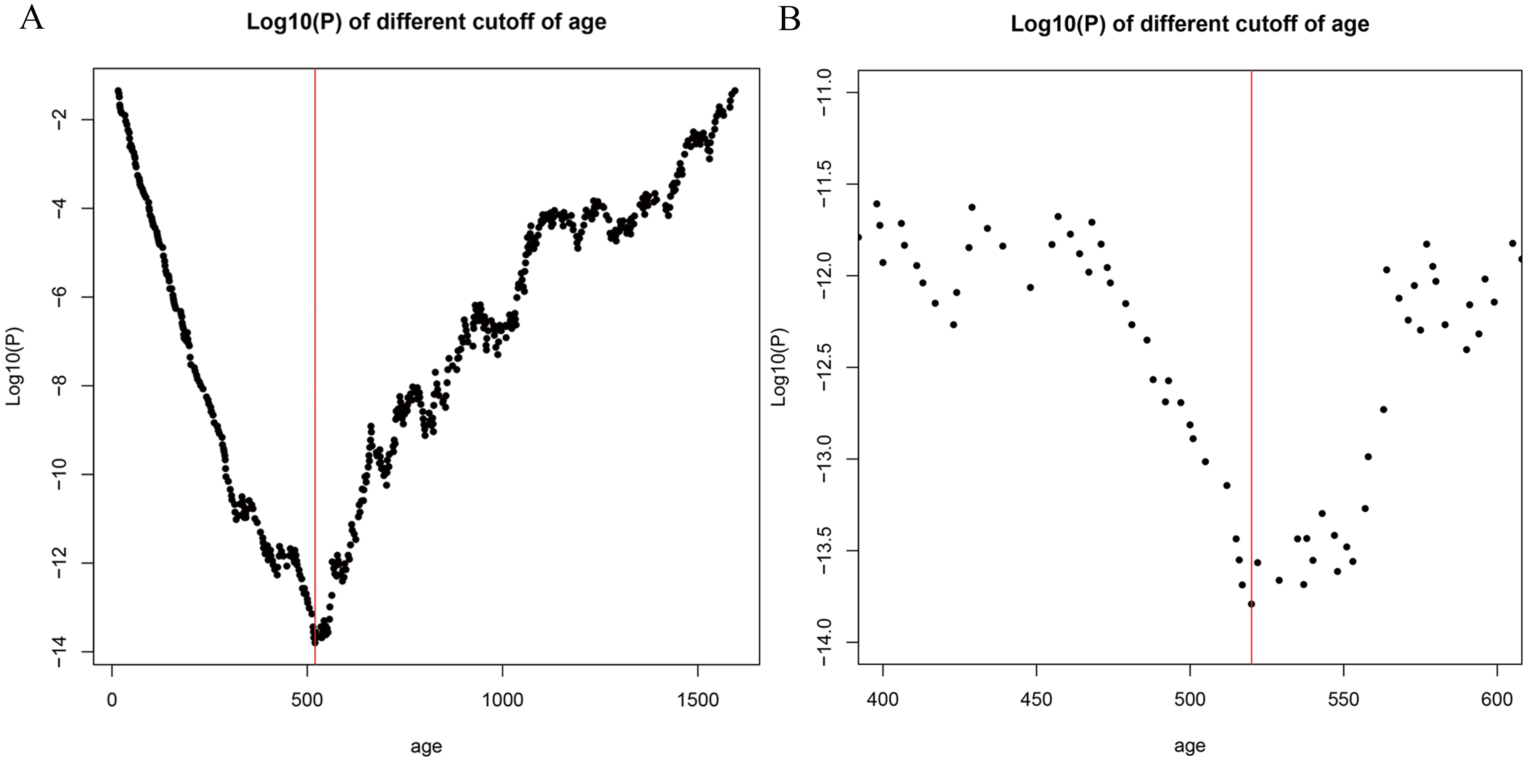

Supplement: Supplemental Information 5 — (A) Log10(P)-age plot. (B) Close-up view of Fig. S2A. [file peerj-07-7316-s005.png]
